# Sensitivity and permissivity of *Cyprinus carpio* to cyprinid herpesvirus 3 during the early stages of its development: importance of the epidermal mucus as an innate immune barrier

**DOI:** 10.1186/s13567-014-0100-0

**Published:** 2014-10-04

**Authors:** Maygane Ronsmans, Maxime Boutier, Krzysztof Rakus, Frédéric Farnir, Daniel Desmecht, Fabien Ectors, Michaël Vandecan, François Lieffrig, Charles Mélard, Alain Vanderplasschen

**Affiliations:** Immunology-Vaccinology, Department of Infectious and Parasitic Diseases (B43b), Fundamental and Applied Research for Animals & Health, Faculty of Veterinary Medicine, University of Liège, 4000 Liège, Belgium; Biostatistics and Bioinformatics applied to Veterinary Science (B43), Fundamental and Applied Research for Animals & Health, Faculty of Veterinary Medicine, University of Liège, 4000 Liège, Belgium; Pathology, Department of Morphology and Pathology (B43), Fundamental and Applied Research for Animals & Health, Faculty of Veterinary Medicine, University of Liège, 4000 Liège, Belgium; Transgenic Platform, Groupe Interdisciplinaire de Génoprotéomique Appliquée, Centre Hospitalier Universitaire B34, University of Liège, 4000 Liège, Belgium; CEFRA-University of Liège, 10 Chemin de la Justice, 4500 Tihange, Belgium; CERgroupe, 1 rue du Carmel, B-6900 Marloie, Belgium

## Abstract

Cyprinid herpesvirus 3 (CyHV-3) causes a lethal disease in common and koi carp (*Cyprinus carpio*). The present study investigated the ability of CyHV-3 to infect common carp during the early stages of its development (from embryos to fingerlings) after inoculation by immersion in water containing the virus. Fish were inoculated at different times after hatching with a pathogenic recombinant CyHV-3 strain expressing luciferase. The sensitivity and permissivity of carp to CyHV-3 were investigated using in vivo bioluminescence imaging. The susceptibility of carp to CyHV-3 disease was investigated by measuring the survival rate. Carp were sensitive and permissive to CyHV-3 infection and susceptible to CyHV-3 disease at all stages of development, but the sensitivity of the two early developmental stages (embryo and larval stages) was limited compared to later stages. The lower sensitivity observed for the early developmental stages was due to stronger inhibition of viral entry into the host by epidermal mucus. In addition, independent of the developmental stage at which inoculation was performed, the localization of light emission suggested that the skin is the portal of CyHV-3 entry. Taken together, the results of the present study demonstrate that carp are sensitive and permissive to CyHV-3 at all stages of development and confirm that the skin is the major portal of entry after inoculation by immersion in infectious water. The results also stress the role of epidermal mucus as an innate immune barrier against pathogens even and especially at the early stages of development.

## Introduction

The common carp (*Cyprinus carpio*) is one of the oldest cultivated fish species. In China, the cultivation of carp dates back to at least the 5^th^ century BC and in Europe, carp farming began during the Roman Empire [1]. Common carp is currently one of the most economically valuable species in aquaculture, as it is one of the main cultivated fish for human consumption with worldwide production of 3.4 million tons per year [2]. Common carp is also produced and stocked in fishing areas for angling purposes. Its colorful ornamental varieties (koi carp), grown for personal pleasure and competitive exhibitions, represent one of the most expensive markets for individual freshwater fish, with some prize-winners being sold for US$10 000–1 000 000 [3].

Carp development comprises successive stages associated with specific morphological and physiological characteristics. The incubation period of fertilized eggs varies between 48 and 72 h depending on water parameters [4,5]. From hatching to 3–5 days post-hatching, fish are called embryos. The digestive tract is not fully developed so the fish rely entirely on the yolk sac as a source of nutrients [6,7]. Because the gills are not yet formed, gas exchange occurs through the blood vessels of the yolk sac and the caudal fin. By 3 to 5 days post-hatching the yolk sac is entirely absorbed and most organs formed and functional. From this moment, the fish are referred to as larvae. In contrast to embryos, larvae rely on exogenous feeding and their respiration is mediated by the gills. Around 2–3 weeks post-hatching, larvae acquire an adult shape and are no longer transparent. When the metamorphosis is complete, the fish are referred to as juveniles. The fish then develop paired fins and maturation of the organs is finalized. Around 35 days post-hatching, the fish are called fingerlings. Fingerlings are fully developed, entirely covered with scales (with the exception of fish that have no scales), and appear just like adults [6,7]. Despite their adult appearance, the immune system of fingerlings is not yet fully mature and functional.

Teleosts develop both innate and adaptive immune responses [8]. During the early stages of life described above, the lymphomyeloid organs of teleosts are not yet fully mature and the fish are unable to develop an effective adaptive immune response. In carp, adaptive immune competency occurs roughly 2 months after hatching [4,9]. Early in life, fish immunity relies on passively transferred maternal factors and on innate immune mechanisms which are activated just after egg fertilization and fully functional at hatching [10,11]. Among the components of the innate immune system, the mucus covering the external and internal surfaces of the fish is thought to play a key role in the inhibition of pathogen entry into the host [8,11].

Koi herpesvirus (KHV), also known as cyprinid herpesvirus 3 (CyHV-3) (genus *Cyprinivirus*, family *Alloherpesviridae*, order *Herpesvirales*), is the etiological agent of an emerging and lethal disease in common and koi carp. Since its emergence in the late 1990s, this highly contagious and dreadful disease has caused severe economic losses in both the common and koi carp culture industries worldwide. Publication of the CyHV-3 sequence [12] and the cloning of its genome as an infectious bacterial artificial chromosome (BAC) [13] allowed the production of CyHV-3 recombinant strains. Recently, we took advantage of these advances to construct a luciferase (LUC)-expressing recombinant strain via intergenic insertion of a LUC expression cassette. Using this recombinant strain and in vivo imaging system (IVIS), we showed that the skin covering the fins and body of carp fingerlings, but not the gills, is the major portal of entry after inoculation by immersion in water containing the virus [14]. Using similar approaches, epidermal mucus has been shown to act as an innate immune barrier, drastically reducing CyHV-3 binding to epidermal cells [15].

The epidermis of adult teleost fish is a living stratified squamous epithelium divided into three layers. The surface and basal layers are single-cell layers composed of keratinocytes and undifferentiated basal cells, respectively. The pluristratified intermediate layer is composed of unicellular glands (mucous cells and club cells), ionocytes and undifferentiated cells. All three layers contain cells capable of mitotic division. The scales are dermal structures covered by the epidermis [16]. Compared to adults, the epidermis of embryos has a simplified structure consisting of surface and basal layers (single-cell layers) interrupted by unicellular glands and ionocytes.

The susceptibility of carp to CyHV-3 disease has been tested at different developmental stages after inoculation by immersion in water containing the virus. Though juveniles, fingerlings, and adults are susceptible to CyHV-3 disease [17-19], larvae have been reported to be resistant to infection based on PCR analysis of experimentally inoculated subjects [19]. These results suggest that the early stages of carp development are resistant to CyHV-3. Interestingly, this resistance could be due to a lack of expression of a component essential for completion of the viral infection (e.g., a cell surface binding receptor) by the early developmental stages and/or the expression of an innate immune mechanism capable of preventing the infection that is not expressed by later development stages.

In the present study, we investigated the ability of CyHV-3 to infect common carp during the early stages of its development (from embryos to fingerlings) after inoculation by immersion in water containing the virus. Fish were inoculated at different times post-hatching with a pathogenic recombinant CyHV-3 strain expressing LUC as a reporter gene. The sensitivity (ability to support viral entry into host cells) and permissivity (ability to support viral replication) of carp to CyHV-3 infection were investigated using IVIS. The susceptibility of carp to CyHV-3 disease was investigated by measuring the survival rate over a period of one month after inoculation. The results of the present study demonstrate that carp are sensitive and permissive to CyHV-3 infection and susceptible to CyHV-3 disease at all developmental stages. However, the fish express reduced sensitivity during the two earlier stages of development (embryo and larval stages) due to efficient inhibition of viral entry into the host by epidermal mucus. This study further supports the importance of the skin as the major portal of entry of CyHV-3 after inoculation by immersion in infectious water and the role of the epidermal mucus as an innate immune barrier against pathogens even and especially during the early developmental stages.

## Materials and methods

### Cells and virus

Common carp brain (CCB) cells [20] were cultured in minimum essential medium (Sigma) containing 4.5 g/L of glucose (D-glucose monohydrate; Merck) and 10% fetal calf serum. The cells were cultured at 25 °C in a humid atmosphere containing 5% CO_2_. CCB cells were used to produce and titrate CyHV-3. The FL BAC revertant ORF136 LUC strain of CyHV-3, hereafter referred to as the FL LUC strain, was described previously [14]. This recombinant strain derived from the FL BAC clone of CyHV-3 encodes a wild type ORF55 locus (in which the BAC cassette was initially cloned) and a firefly (*Photinus pyralis*) luciferase expression cassette under control of the human cytomegalovirus immediate early promoter. The LUC cassette was inserted in the intergenic region between open reading frames ORF136 and ORF137.

### Carp reproduction

Common carp (*Cyprinus carpio*) were produced by artificial reproduction. Breeders free of CyHV-3 based on PCR analysis of peripheral mononuclear blood cells and serological analysis that generated offspring sensitive to CyHV-3 disease were hosted in a recirculating aquaculture system at 23 °C (CEFRA, University of Liège, Belgium). Mature breeders were selected based on morphological characteristics. Ovulation and spawning were induced by two intraperitoneal (IP) injections of Ovaprim (Syndel) performed at an 8 h interval. The first injection was a dose of 0.05 mL/kg of body weight. The dose of the second injection differed between males and females: 0.25 and 0.5 mL/kg of body weight, respectively. Eggs were stripped 12 h after the second hormonal injection and fertilized with stripped sperm. All manipulations of the breeders were performed under anesthetic tranquilization with benzocaine (50 mg/L of water). Fertilized eggs were incubated in Zoug bottles until hatching. Embryos (mean body weight 1.2 mg) were transferred to a 50 L tank (2500 larvae per tank). The water temperature was regulated at 24 °C and the O_2_ concentration maintained above 6 ppm. Larvae were fed *ad libitum* with artificial larval food Gemma micro (Skretting). The food was distributed manually 6 times per day during the first 45 days post-hatching. Juveniles reached a mean body weight of 500 mg within their first month of life.

### CyHV-3 inoculation of fish

For viral inoculation mimicking natural infection, fish were kept for 2 h in water containing the FL LUC strain of CyHV-3 (400 plaque-forming units (pfu)/mL). At the end of the incubation period, the fish were returned to 50 L tanks where they were kept in floating breeding nests (SERA). To avoid removal of the epidermal mucus, fish were caught using a container rather than a fish net. In some experiments, the skin mucus was partially removed before inoculation. Carp were caught with a fish net, placed on a plastic sheet, and the upper side of the body was gently rubbed with a floqswab ultrathin (Copan flock technologies, Brescia, Italy) to remove the epidermal mucus. Carp were also inoculated by IP injection as follows. Carp were anesthetized by immersion in water containing benzocaine (25 mg/L) and distributed individually to the wells of a macroscopic slide (12 cavity microscopic slide, VWR international) containing a viscous solution (NaCl 292 mg/L, KCl 12.6 mg/L, CaCl_2_.2H_2_O 48.3 mg/L, MgSO_4_.7H_2_O 81.6 mg/L, methylene blue 1 mg/L, carboxyl methyl cellulose high viscosity 20 g/L). Culture medium containing 3.6 × 10^6^ pfu/mL of the FL LUC strain was injected intraperitoneally (corresponding to an approximate dose of 40 pfu per fish) using a microinjector (Femtojet, Eppendorf, VWR international) and tapered beveled pipettes (15–20 μm). Injection was performed using the following parameters, leading to the administration of an approximate volume of 0.01 μL: automatic mode, injection pressure of 200 hPa, compensation pressure of 0 hPa, and injection time of 0.2 - 0.8 s according to fish size. After injection, the fish were returned to 50 L tanks. This animal study was accredited by the local ethics committee of the University of Liège.

### In vivo bioluminescence imaging

Imaging of firefly LUC expression was performed using IVIS (IVIS spectrum, Xenogen, Caliper LifeSciences) as described previously [14,15,21]. D‑luciferin (Xenogen, Caliper LifeSciences) (15 mg/mL) in phosphate-buffered saline (PBS) was microinjected into the peritoneal cavity as described above. Efficient injection of D‑luciferin into fish was controlled by examination of the fish using full field epifluorescent microscopy (Eclipse TE2000-S, Nikon). D‑luciferin emits a green fluorescence when excited with a blue light (488 nm). After an incubation period of 10 min, the carp were analyzed by IVIS. Images were collected using the following settings: field of view A, binning on small, automatic exposure time with a maximum of 1 min, and a subject height of 0.35 cm. The relative intensities of transmitted light from bioluminescence were represented as a pseudocolor image ranging from violet (least intense) to red (most intense). For quantitative comparisons, regions of interest were manually drawn by surrounding the fish outline, and the Living Image software 3.2 (Caliper Life Sciences) was used to calculate the corrected average radiance (p/s/cm^2^/sr) by subtracting the average radiance of the background for each image.

### Histological analysis

Euthanized fish were fixed by immersion in Carnoy solution (ethanol:acetic acid:chloroform 6:1:3, v/v/v) for 24 h at 4 °C. After dehydration with ethanol, samples were embedded in paraffin blocks [15]. Five micron sections were stained with a combined Periodic Acid-Schiff, Alcian Blue and hematoxylin-eosin staining prior to microscopic analysis using a Leica DM 2000 LED microscope equipped with a DFC 450C camera (Leica, Heerbrugg, Switzerland).

### Statistical analysis

Significant differences in the log of the average radiance emitted by IVIS-positive fish, according to time of inoculation (days post-hatching) and time post-inoculation or according to time of inoculation and mode of inoculation, were assessed using two-way ANOVA with interactions. Significant differences in the number of IVIS-positive fish according to time of inoculation (days post-hatching), time post-inoculation and mode of inoculation were assessed using logistical analysis and the chi-squared test. Significant differences in survival rates according to time of inoculation (days post-hatching) were assessed using a contingency table and chi-squared test.

## Results

The goal of the present study was to investigate the ability of CyHV-3 to infect *Cyprinus carpio* during the early stages of its development. The data presented in this manuscript are representative of duplicate independent experiments. Importantly, though the different experiments were initially performed successively, the data set presented in this manuscript represents the parallel repetition of all experiments with the same batch of fish.

### Sensitivity and permissivity of *Cyprinus carpio* to CyHV-3 during the early stages of development

**Figure 1:**
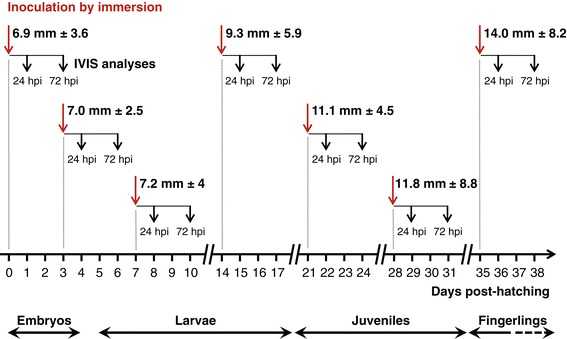
**Timeline of the experiments performed to investigate the sensitivity and permissivity of carp to CyHV-3 during the early stages of development.** At different times post-hatching (indicated by red arrows), carp were inoculated by immersion in water containing the FL LUC strain. At 24 and 72 hpi, 30 carp were analyzed individually by IVIS. The two-headed arrows below the time scale represent the period of time during which the indicated developmental stage represented at least 80% of the fish population. The size of fish (mean ± SD, based on 15 measurements) at the time of inoculation (indicated by vertical red arrows) is indicated.

**Figure 2:**
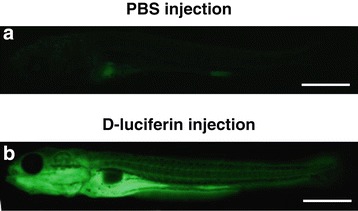
**Quality control of D‑luciferin microinjection by epifluorescent microscopy.** Prior to IVIS analysis, D‑luciferin was microinjected into the peritoneal cavity of carp. The success of the injection was controlled by epifluorescent microscopy of the subjects with D‑luciferin autofluorescence. The upper and lower panels represent fish injected with PBS **(panel a)** and D‑luciferin **(panel b)**, respectively. Both subjects illustrating the larval stage (15 days post-hatching) were examined under full field 488 nm excitation. Scale bars = 1 mm.

**Figure 3:**
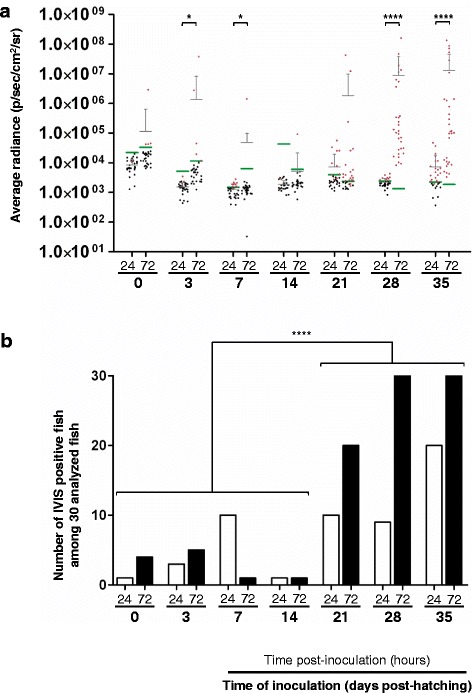
**Quantitative measurements of the sensitivity and permissivity of carp to CyHV-3 during the early stages of development.** Fish were mock-inoculated or inoculated at the indicated times post-hatching and then analyzed by IVIS at 24 and 72 hpi. **(a)** The average radiance (p/sec/cm^2^/sr) emitted by individual infected fish (*n* = 30 per group) corrected for the background of each image is presented by dots. For each group of inoculated fish (according to time of inoculation and time post-inoculation), the mean + SD is presented as grey bars. For each time point, a group of mock-inoculated fish was analyzed to define the threshold of positivity (green bars), defined as the mean + 3 SD (*p* < 0.00135). Red dots represent infected fish defined as positive based on their average radiance being higher than the threshold or based on the expression of a spot of light (even if their average radiance was below the threshold). The data obtained at different times post-inoculation (24 versus 72 hpi) for each time of inoculation were compared by two-way ANOVA. **(b)** The number of positive fish among 30 analyzed inoculated fish. The number of positive fish according to time of inoculation was compared by logistical analysis. Inoculations performed at the embryonic and larval stages (between 0 and 14 days post-hatching) were compared to inoculations at the post-larval stages (between 21 and 35 days post-hatching). *p < 0.05, ****p < 0.0001.

**Figure 4:**
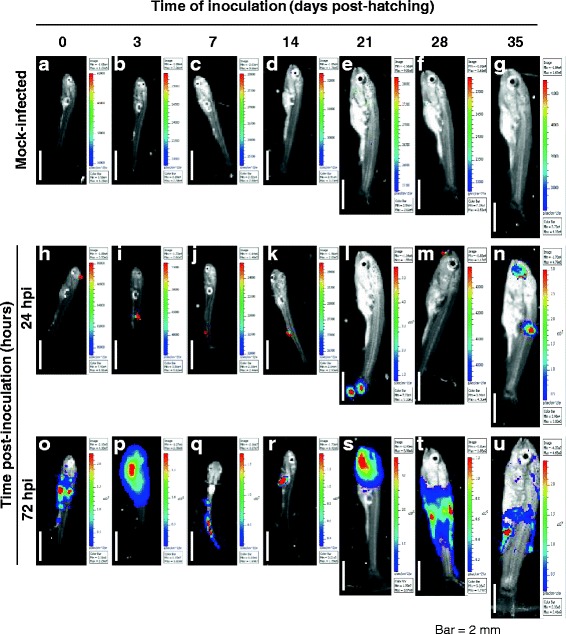
**IVIS images of the sensitivity and permissivity of carp to CyHV-3 during the early stages of development.** The fish inoculation methods and IVIS analysis are described in the legend of Figures 1 and 3. Mock-infected fish **(panels a to g)** and representative positive infected fish **(panels h to u)** as described in Figure 3 are shown for each time point of analysis. Images are presented with a relative photon flux scale automatically adapted to each image in order to use the full dynamic range of the pseudocolor scale. Scale bars = 2 mm.

Figure 1 presents the design of the experiment performed to address the sensitivity and permissivity of the early developmental stages of common carp to CyHV-3 after inoculation by immersion in infectious water. Carp were inoculated at different times post-hatching that are representative of the different developmental stages: days 0 and 3 for the embryo stage, days 7 and 14 for the larval stage, days 21 and 28 for the juvenile stage, and day 35 for the fingerling stage. At 24 and 72 h post-inoculation (hpi), fish were injected intraperitoneally with D-luciferin. The small size of the earlier stages of development made the injection of D‑luciferin a critical step. Taking advantage of the autofluorescence of D‑luciferin, the success of the injection was controlled by full field epifluorescent examination of each fish prior to IVIS analysis (Figure 2). In contrast to fish injected with PBS, fish successfully injected with D‑luciferin expressed green fluorescence in the peritoneal cavity and vascularized tissues. Twenty-four and 72 h after each inoculation time point (0, 3, 7, 14, 21, 28, and 35 days post-hatching), 30 successfully injected fish were analyzed using the IVIS (Figures 3 and 4). The threshold of positivity was determined based on the analysis of mock-inoculated fish (mean plus 3 standard deviations (SD), *p* < 0.00135; Figure 3). Fish with an average radiance higher than the threshold were classified as positive. Examination of IVIS images revealed that the vast majority exhibited at least one focal source of light. Examination of IVIS images from both the mock-inoculated and inoculated groups that had an average radiance lower than the threshold of positivity revealed only a few fish, exclusively in the inoculated groups, that expressed a focal source of light. Consequently, these fish were also classified as positive (Figure 3a, see red dots below the threshold represented by a green bar). Images illustrating such a positive fish with an average radiance below the threshold are presented in Figure 4 (see panels h, i, and k). Interestingly, examination of images from all positive fish revealed that spots of light were distributed randomly on the body, head, and fins. On one occasion, a LUC signal was observed on the eye ball (Figure 4, panel n). None of the spots had a position compatible with the gills. These data suggest that, independent of the developmental stage at which the inoculation was performed, the skin is the major portal of entry after inoculation by immersion in water containing the virus.

Independent of the developmental stage at the time of inoculation and the time of analysis post-inoculation (24 or 72 hpi), at least one positive fish was detected (Figure 3), demonstrating that carp are sensitive to CyHV-3 infection at all stages of development. Photon emission was significantly higher 72 hpi compared to 24 hpi for the following times of inoculation (Figure 3A): 3 (*p* < 0.05), 7 (*p* < 0.05), 28 (*p* < 0.0001), and 35 (*p* < 0.0001) days post-hatching. These data suggest that carp are permissive to CyHV-3 at all stages of development, which was further supported by the IVIS images (Figure 4). Compared to images collected 24 hpi, those collected at 72 hpi systematically revealed more spots of light with a larger area and greater maximum radiance independent of the time of inoculation (Figure 4, second vs. third rows of panels).

These data demonstrate that all developmental stages of carp are sensitive and permissive to CyHV-3 infection. However, the percentage of positive fish was significantly lower after inoculation 0 – 14 days post-hatching compared to inoculation at later stages (Figure 3b, *p* < 0.0001). No significant difference was found in the average radiance (p/sec/cm^2^/sr) of positive fish (independent of the stage at which inoculation was performed) (*p* < 0.05), implying that, once infected, replication occurs similarly in fish, at least at the portal of entry.

### Effect of epidermal mucus on the sensitivity of *Cyprinus carpio* to CyHV-3 during the early stages of development

**Figure 5:**
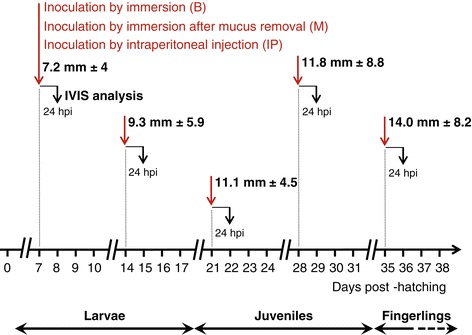
**Timeline of the experiments performed to investigate the effect of the mode of inoculation on the sensitivity of carp to CyHV-3 infection during the early stages of development.** At different times post-hatching (indicated by red arrows), carp were inoculated with the FL LUC strain according to three modes of inoculation: by immersion in infectious water (B), by immersion in infectious water just after removing the epidermal mucus (M), and by IP injection of the virus (IP). At 24 hpi, 30 carp were analyzed individually by IVIS. The two-headed arrows below the time scale represent the period of time during which the indicated developmental stage represented at least 80% of the fish population. The size of fish (mean ± SD, based on 15 measurements) at the time of infection (indicated by vertical red arrows) is indicated.

**Figure 6:**
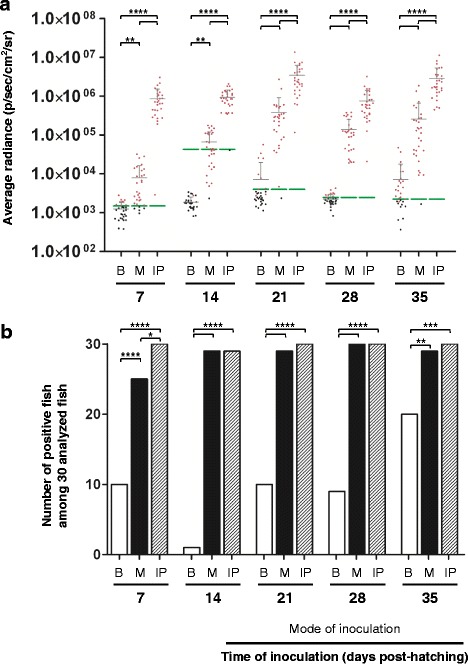
**Quantitative measurements of the effect of the mode of inoculation on the sensitivity of carp to CyHV-3 infection during the early stages of development.** The timeline of this experiment has been described in Figure 5. Fish were mock-inoculated or inoculated with the FL LUC strain using three modes of infection: immersion in infectious water (B), immersion in infectious water just after removing the epidermal mucus (M), and by IP injection of the virus (IP). Fish were analyzed by IVIS at 24 hpi. **(a)** The average radiance (p/sec/cm^2^/sr) emitted by individual infected fish (*n* = 30 per group) corrected for the background of each image is presented by dots. For each group of inoculated fish (according to time of inoculation and mode of inoculation), the mean + SD is presented as grey bars. For each time point, a group of mock-inoculated fish was analyzed to define the threshold of positivity (green bars), defined as the mean + 3 SD (*p* < 0.00135). Red dots represent inoculated fish defined as positive based on their average radiance being higher than the threshold or based on the expression of a spot of light (even if their average radiance was below the threshold). The data obtained for the different modes of inoculation for a considered time of inoculation were compared by two-way ANOVA. **(b)** The number of positive fish among 30 analyzed infected fish. The data obtained for the different modes of inoculation for each time of inoculation were compared using the chi-squared test. *p < 0.05, **p < 0.01, *** *p* < 0.001, and ****p < 0.0001.

**Figure 7:**
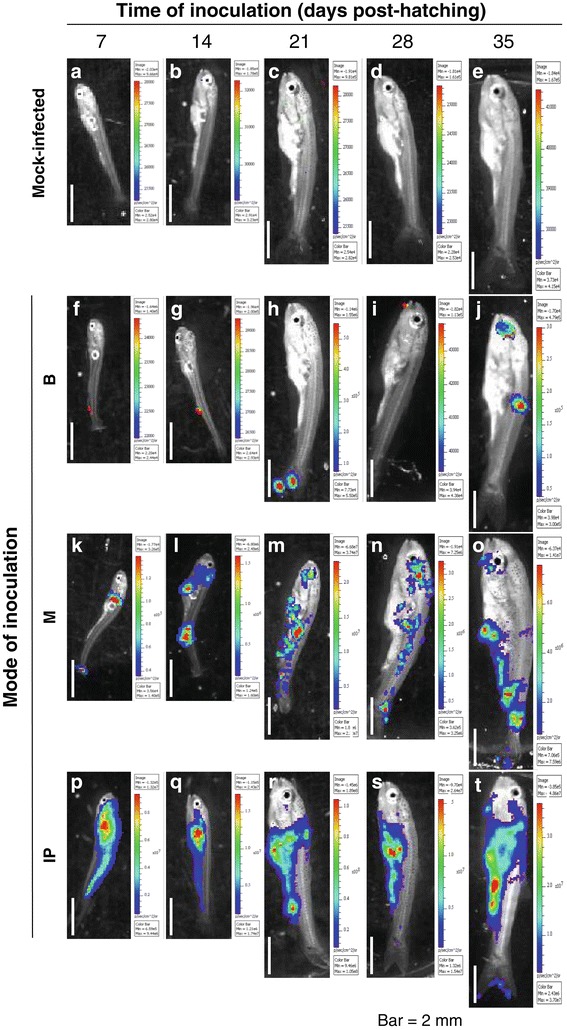
**IVIS images of the effect of the mode of inoculation on the sensitivity of carp to CyHV-3 during the early stages of development.** The fish inoculation methods and IVIS analysis are described in the legend of Figures 5 and 6. Mock-infected fish **(panels a to e)** and representative positive infected fish **(panels f to t)** as described in Figure 6 are shown for each time point of analysis. Images are presented with a relative photon flux scale automatically adapted to each image in order to use the full dynamic range of the pseudocolor scale. Scale bars = 2 mm.

Taken together, the data demonstrate that the embryo and larval stages are less sensitive to CyHV-3 infection than the juvenile and fingerling stages. The lower sensitivity of these early stages of development could be due to an absence of host expression of a component essential for completion of the viral infection (e.g., a cell surface binding/entry receptor) and/or the expression of an innate immune mechanism capable of preventing infection. Recently, we demonstrated that epidermal mucus acts as an innate immune barrier against CyHV-3 entry in fingerlings and adults [15]. In the present study, we investigated whether the lower sensitivity observed for the early developmental stages can be explained by a higher anti-viral activity of the epidermal mucus. To test this hypothesis, the different developmental stages of carp were inoculated using three different modes of inoculation (Figure 5): (i) immersion in water containing the virus (Bath, B), (ii) immersion in infectious water after removal of epidermal mucus (Mucus, M), and (iii) IP injection (IP). Inoculation times had to be restricted to at least 7 days post-hatching because younger fish did not support the stress induced by the process of mucus removal or IP inoculation. Independent of the stage at which IP inoculation was performed, nearly all inoculated fish expressed an intense and comparable LUC signal 24 hpi (Figure 6a). Visual examination of IVIS images revealed that fish inoculated intraperitoneally expressed an intense signal throughout the peritoneal cavity. This observation further supports the hypothesis that the lower sensitivity observed for early developmental stages is the consequence of a restriction of viral entry at the portal of entry. Some signals located on the fins likely reveal skin contamination that occurred during the IP inoculation (Figure 7, see panels s and t).

**Figure 8:**
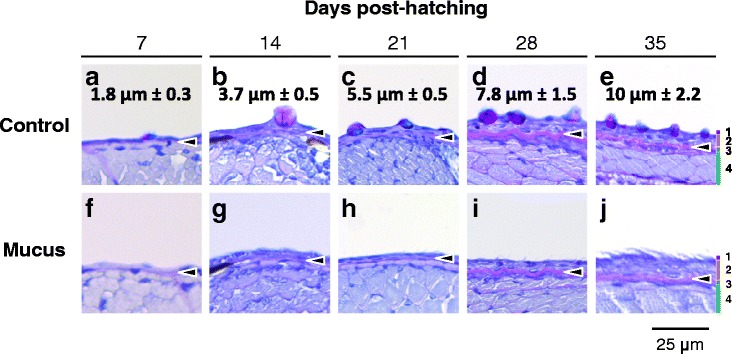
**Histological structure of skin epidermis during the early stages of carp development.** At the indicated days post-hatching, carp were harvested and either left untreated (Control, **panels a to** **e**) or treated for removal of the epidermal mucus (Mucus, **panels f to** **j**). The fish were then euthanized and processed for histological examination of the skin epidermis. The numbers in the upper panels represent the thickness of the epidermis (mean ± SD, based on 15 measurements). Histological structures were identified for the samples collected 35 days post-hatching **(panels e and j)**: 1, mucus; 2, epidermis; 3, basement membrane (arrow head); 4, dermis and dorsal muscles. Scale bar = 25 μm.

Independent of the developmental stage tested, removal of the epidermal mucus prior to inoculation by immersion in infectious water drastically increased the sensitivity of carp to CyHV-3. Fish inoculated by immersion after the removal of mucus had a significantly higher LUC signal than untreated control fish (Figure 6, compare M and B). In terms of the number of positive fish, removal of mucus prior to inoculation led to the percentage of positive fish being close to maximum and similar to that observed after IP inoculation (with the exception of inoculations performed 7 days post-hatching), and systematically higher than those observed for control inoculated fish (Figure 6b). The IVIS images presented in Figure 7 demonstrate that fish inoculated after mucus removal expressed more spots of light with a larger area and higher intensity than control infected fish (Figure 7, compare the second and third rows of panels). Finally, we controlled the effect of the treatment to remove the mucus by histological examination of the skin epidermis (Figure 8). Despite the use of fixation conditions developed for visualization of the mucus, the mucus layer covering the surface of the epidermis was barely visible in some slides (Figure 8, upper panels). However, unicellular mucous glands containing mucus were easily identified throughout the epidermis at all stages of development. In contrast, these cells were no longer identifiable when the mucus was removed. The effect of the treatment was also visible on the surface of the uppermost layer of epidermal cells, which had a hairy appearance (Figure 8, panels h-j). The observed evolution of the structure of the epidermis according to ontogenesis was consistent with an earlier report [16].

Taken together, the results demonstrate that the lower sensitivity to CyHV-3 observed for the early developmental stages of carp is due at least in part to a strong inhibition of viral entry into the host by the epidermal mucus.

### Susceptibility of *Cyprinus carpio* to CyHV-3 disease during the early stages of development

Carp were mock-inoculated or inoculated with the FL LUC strain at the indicated times post-hatching. Survival rates were measured over a period of 30 days post-inoculation (dpi). For most groups, surviving fish were counted on days 15 and 30 post-inoculation.

**Figure 9:**
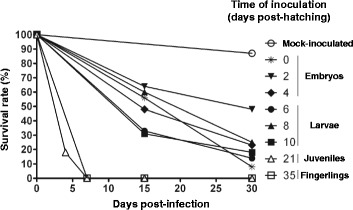
**Susceptibility of** ***Cyprinus carpio*** **to CyHV-3 disease during the early stages of its development.** Carp (*n* = 80) were mock-inoculated or inoculated with the FL LUC strain at the indicated times post-hatching by immersion in infectious water. Survival rates were measured over a period of 30 days post-infection.

The data were analyzed as follows. First, the survival rate of each infected group was compared to the control mock-inoculated group. Independent of the age at which the inoculation was performed or the time post-inoculation at which the survival rate was measured, each infected group had a survival rate lower than the control mock-inoculated group (*p* < 0.0001 at 15 and 30 dpi). Second, statistical analyses were performed after grouping the data related to the developmental stages at which inoculation was performed (Figure 9) as follows: embryo-larval stage (fish inoculated from 0 to 10 days post-hatching), juvenile stage (fish inoculated at day 21), and fingerling stage (fish inoculated at day 35). These analyses demonstrate that the mortality rate was significantly different between the three developmental stages tested at 15 (*p* < 0.0001) and 30 dpi (*p* < 0.0001), but mainly between the embryo-larval and two older stages.

## Discussion

The goal of the present study was to investigate the ability of CyHV-3 to infect common carp during the early stages of its development (from embryos to fingerlings) after inoculation by immersion in infectious water. Using IVIS, we demonstrated that carp are sensitive and permissive to CyHV-3 infection at all stages of development. However, the sensitivity of the two early stages (embryo and larval stages) was limited compared to the older stages (juvenile and fingerling stages). Inoculation after removal of the epidermal mucus demonstrated that the reduced sensitivity of the early developmental stages was caused by a stronger inhibition of viral entry into the host by the epidermal mucus. Finally, the susceptibility of carp to CyHV-3 disease was tested by inoculating carp at different times after hatching and measuring the survival rate over a period of one month after inoculation. The data are consistent with the conclusion that carp are susceptible to CyHV-3 at all stages of development and that the sensitivity to CyHV-3 infection increases during ontogenesis.

Ito et al. reported that the larvae from two independent strains of common carp were resistant to CyHV-3 infection but juveniles from the same strains were highly susceptible [19]. The results of the present study contradict this earlier report by demonstrating that carp are sensitive and permissive to CyHV-3 at all stages of development. Importantly, the results of the present study are representative of two experiments performed with unrelated breeders. All of the breeders used in this study were proved to be free of CyHV-3. In their study, Ito et al. did not mention whether they controlled the serological status of the carp used for reproduction. One interesting hypothesis that could explain the paradox between the two studies is that the previous study used seropositive genitors. These immune genitors could have transferred transient humoral immune protection against CyHV-3 to their offspring. Teleost fish lay telolecithal eggs in which passive transfer of maternal antibodies occurs [22,23]. Whether embryos from immune versus naive breeders exhibit different sensitivity to CyHV-3 would be interesting to determine in the future, as well as how long this passive protection lasts. Experiments to test this hypothesis are currently in progress.

Epidermal mucus acts as an innate immune barrier against CyHV-3 entry into the host [15]. The results of the present study suggest that the lower sensitivity to CyHV-3 of the early developmental stages is due to more efficient protection by the mucus, which is conferred through two types of complementary mechanisms. First, the mucus forms an efficient mechanical barrier that constantly moves downstream along the fish and off of the trailing edges. Similar to the muco-ciliary escalator of the respiratory tract of pulmonate animals, fish mucus reduces pathogen access to epithelial cells. Second, the mucus contains numerous proteins, such as immunoglobulins, enzymes, and lytic agents, capable of neutralizing microorganisms [24-28]. Several hypotheses, which are not mutually exclusive, could explain the more efficient inhibition of CyHV-3 entry by the epidermal mucus in the early developmental stages. One hypothesis is that the mucus layer of the early stages of development forms a more uniform mechanical barrier on the fish body, possibly due to its biochemical composition, the hydrodynamic parameters of the fish, or as a consequence of reduced physical interactions between the fish and an object or other fish [29]. Another hypothesis is that the mucus produced by early stages of development contains a higher concentration of biologically active molecules capable of neutralizing CyHV-3. Even if technically difficult, it would be very interesting to compare the neutralization activity of soluble mucus extracts from larvae versus older stages that are more sensitive to CyHV-3. These experiments could reveal that the early stages of development express biologically active innate immune molecules in their mucus that are not expressed, or expressed at lower levels at later stages. In addition to their interest to fundamental science, such conclusions could be very interesting for application-oriented research, such as that trying to enhance fish resistance against pathogens by up-regulating innate immunity.

In conclusion, the present study demonstrates that carp are sensitive and permissive to CyHV-3 infection at all stages of development, even if the sensitivity of the early stages is reduced due to efficient inhibition of viral entry by the epidermal mucus. This study further supports the importance of the skin as the major portal of entry of CyHV-3 after inoculation by immersion in infectious water. It stresses the role of the epidermal mucus as an innate immune barrier against pathogen even and especially during the early stages of development.

## Abbreviations

BAC: Bacterial artificial chromosome
CCB: Common carp brain
CyHV-3: Cyprinid herpesvirus 3
dpi: days post-infection
hpi: hours post-infection
IP: Intraperitoneal
IVIS: In vivo imaging system
KHV: Koi herpesvirus
LUC: Luciferase gene
PBS: Phosphate-buffered saline
pfu: plaque-forming unit
pi: post-infection
p/s/cm^2^/sr: photon/second/centimeter square/steradian
SD: Standard deviation
